# The Relationship between the Second-to-Fourth Digit Ratio and Behavioral Sexual Dimorphism in School-Aged Children

**DOI:** 10.1371/journal.pone.0146849

**Published:** 2016-01-12

**Authors:** Takahiko Mitsui, Atsuko Araki, Chihiro Miyashita, Sachiko Ito, Tamiko Ikeno, Seiko Sasaki, Takeya Kitta, Kimihiko Moriya, Kazutoshi Cho, Keita Morioka, Reiko Kishi, Nobuo Shinohara, Masayuki Takeda, Katsuya Nonomura

**Affiliations:** 1 Department of Urology, University of Yamanashi Graduate School of Medical Science, Chuo-city, Japan; 2 Department of Urology, Hokkaido University Graduate School of Medicine, Sapporo, Japan; 3 Hokkaido University Center for Environmental and Health Sciences, Sapporo, Japan; 4 Department of Public Health, Hokkaido University Graduate School of Medicine, Sapporo, Japan; 5 Department of Obstetrics and Gynecology, Hokkaido University Graduate School of Medicine, Sapporo, Japan; 6 Department of Urology, Kushiro Rosai Hospital, Kushiro, Japan; University of Tuebingen Medical School, GERMANY

## Abstract

Sexually dimorphic brain development and behavior are known to be influenced by sex hormones exposure in prenatal periods. On the other hand, second-to forth digit ratio (2D/4D) has been used as an indirect method to investigate the putative effects of prenatal exposure to androgen. In the present study, we herein investigated the relationship between gender-role play behavior and the second-to-fourth digit ratio (2D/4D), which has been used as an indirect method to investigate the putative effects of prenatal exposure to androgens, in school-aged children. Among 4981 children who became 8 years old by November 2014 and were contactable for this survey by The Hokkaido Study of Environment and Children's Health, 1631 (32.7%), who had data for 2D/4D and Pre-school Activities Inventory (PSAI) as well as data for the survey at baseline, were available for analysis. Parents sent reports of PSAI on the sex-typical characteristics, preferred toys, and play activities of children, and black and white photocopies of the left and right hand palms via mail. PSAI consisted of 12 masculine items and 12 feminine items, and a composite score was created by subtracting the feminine score from the masculine score, with higher scores representing masculine-typical behavior. While composite scores in PSAI were significantly higher in boys than in girls, 2D/4D was significantly lower in boys than in girls. Although the presence or absence of brothers or sisters affected the composite, masculine, and feminine scored of PSAI, a multivariate regression model revealed that 2D/4D negatively correlated with the composite scores of PSAI in boys, whereas no correlation was found in girls. Although 2D/4D negatively correlated with the masculine score in boys and girls, no correlation was observed between 2D/4D and the feminine score. In conclusion, although social factors, such as the existence of brother or sisters, affect dimorphic brain development and behavior in childhood, the present study revealed that the prenatal hormonal environment was an important factor influencing masculine-typical dimorphic brain development and behavior in school-aged children.

## Introduction

Sexual dimorphism is characterized by differences in cognitive functions between boys and girls, and has been associated with anatomical and physiological differences in the brain. Sexually dimorphic brain development and behavior are known to be influenced by exposure to sex hormones during prenatal periods. In humans, androgen levels increase between 8 and 24 weeks of gestation in boys, and this early androgen exposure has been suggested to affect masculine brain development and behavior[1, 2]. Conversely, the brain develops in a feminine manner in the absence of androgens. Girls with congenital adrenal hyperplasia (CAH), who have some degree of genital ambiguity due to androgen exposure during the prenatal period, display more male-typical behaviors[3–6]. XY-chromosome individuals with complete androgen insensitivity syndrome (CAIS) show fewer male-typical behaviors and more female-typical behaviors despite normally functioning testes with androgen exposure during gestation[7, 8]. These findings have been supported by studies using animals[9–11], which revealed that experimental manipulations of hormones during gestation affected the sexual dimorphism of behaviors.

The second-to-fourth digit ratio (2D/4D) has been used as an indirect method to investigate the putative effects of prenatal exposure to androgens, and was previously reported to be smaller in males than in females[12, 13]. This sex difference in digits has been linked to the prenatal hormonal environment through androgen receptors located in fetal cartilaginous tissue[14], and this tentative theory is supported by the following findings; lower 2D/4D in girls with CAH[15], higher 2D/4D in individuals with CAIS[16], and a relationship between 2D/4D and polymorphisms in androgen receptors[17]. We previously reported that 2D/4D in school-aged children was affected by prenatal Leydig cell function in boys[13].

The present study focused on the hypothesis that sexually dimorphic brain development and behavior are associated with sexual differences in digits, which are affected by the prenatal hormonal environment, particularly androgen exposure. In the present study, we investigated the relationship between gender-role play behavior and 2D/4D in school-aged children.

## Methods

### Participants

This prospective birth cohort study was based on the Hokkaido large-scale cohort, The Hokkaido Study on Environment and Children’s Health[18, 19]. Study details regarding the population, data collection, sampling of biological specimens, and contents of the questionnaire have been described previously[18, 19]. Briefly, native Japanese women living in Hokkaido prefecture were enrolled in this study at <13 weeks of gestation at 37 hospitals and clinics in Hokkaido prefecture between February 2003 and March 2012. Of 20929 pregnant women registered to The Hokkaido Study of Environment and Children's Health, survey sheets including Pre-school Activities Inventory (PSAI) and a request letter for photocopies of both hand palms were sent to 4981 children, who became 8 years old by November 2014 and were contactable for this survey. A total 1631 out of 4981 children (32.7%), who had data for 2D/4D and PSAI as well as data for the survey at baseline, were available for analysis. In these 1631 children, data for the survey at baseline, children at birth, 2D/4D, and PSAI were available for analysis.

This study was approved by the Institutional Ethical Board for Epidemiological Studies at Hokkaido University Graduate School of Medicine and the Hokkaido University Center for Environmental and Health Sciences. All participants provided written informed consent. Written informed consent on behalf of the children enrolled was provided by their parents.

### Outcomes

Primary outcome of the present study was relationship between PSAI and 2D/4D analyzed by a multivariate analysis. Further, secondary outcomes were as following; relationship between PSAI and 2D/4D analyzed by a univariate analysis and relationship between PSAI and the existence of brother or sisters analyzed by univariate and multivariate analyses.

### Gender role behavior using PSAI (Table 1)

PSAI, which is a questionnaire in which parents indicate their child’s involvement in various sex-type behaviors[20, 21], was used to assess gender role behavior in children. We used the Japanese version of PSAI, which was validated from a translation of the English version of PSAI with confirmed retranslation[22]. PSAI included 24 items grouped into the following three categories; preferred toys, preferred activities, and behavioral characteristics. Each item had a score of 1 to 5, representing the response categories: never, hardly ever, sometimes, often, and very often. PSAI consisted of 12 masculine items and 12 feminine items. The composite score in PSAI was calculated by the following formula: 47.11 + 1.1 x (masculine score–feminine score) (Table 1). This formula was derived from the previous study for Japanese participants by Sasaki et al. [22], and we also confirmed that this formula could be applicable to the population of the present study. Higher composite scores represented masculine-typical behavior.

**Table 1:** Preschool Activities Inventory (PSAI).

Part 1: Toys	Please answer these questions according to how often the child played with the following toys during the past month.
1	Guns (or used objects as guns)
2	*Jewellery*
3	Tool set
4	*Dolls*, *doll’s clothes or doll’s carriage*
5	Trains, cars or airplanes
6	Swords (or used objects as swords)
7	*Tea set*
Part 2: Activities	Please answer these questions according to how often the child engaged in the following activities during the past month.
1	*Playing house (e*.*g*. *cleaning*, *cooking)*
2	*Playing with girls*
3	*Pretending to be a female character (e*.*g*. *princess)*
4	Playing at having a male occupation (e.g. solider)
5	Fighting
6	*Pretending to be a family character (e*.*g*. *parent)*
7	Sports and ball games
8	Climbing (e.g. fences, trees, gym, equipment)
9	*Playing at taking care of babies*
10	Showing interest in real cars, trains and airplanes
11	*Dressing up in girlish clothes*
Part 3: Characteristics	Please answer these questions according to how often the child shows the following characteristics.
1	Likes to explore new surroundings
2	Enjoys rough and tumble play
3	Shows interest in snakes, spiders or insects
4	*Avoids getting dirty*
5	*Likes pretty things*
6	*Avoids taking risks*

Regular formatting: masculine score, *Italic* formatting: feminine score

### Measurement of 2D/4D

Children were requested via a mail to send black-and-white photocopies of the palms of the left and right hands. Measurements of digits were made from photocopies of the ventral surfaces of the right and left hands. The participants were instructed to straighten their fingers and lightly place their hands palm down on the photocopy machine. Measurements were made to the nearest 0.5 mm from the mid-point of the finger crease proximal to the palm to the tip of the finger using steel Vernier calipers. The ratio was calculated by dividing the length of the second digit by that of the fourth digit[12]. All measurements were taken twice by two observers blinded to participants’ information in order to confirm the measurements obtained, as previously described[13].

### Effects of brothers or sisters on PSAI

Social factors, particularly the existence of brothers and/or sisters, have been suggested to affect dimorphic brain development and behavior in children. The effects of brothers or sisters on PSAI were also separately investigated using data for the survey.

### Statistical analyses

Data on the characteristics of participants, PSAI, and 2D/4D were presented as the mean ± standard deviation and were analyzed between groups using a one-way ANOVA. The relationship between PSAI and 2D/4D was calculated using Spearman’s rank-order correlation and a multiple linear regression analysis. Further, the relationship between PSAI and the existence of brother or sisters was also calculated using Spearman’s rank-order correlation and a multiple linear regression analysis. The inclusion of covariates was based on biological considerations and adjustments were made for birth weight (continuous), maternal smoking during pregnancy (yes or no), maternal alcohol consumption during pregnancy (yes or no), the presence or absence of brothers (yes or no), and the presence or absence of sisters (yes or no). All statistical analyses were performed using JMP pro 10 (SAS institute Inc., NC, USA). Significance levels were set to 0.05 for all comparisons.

## Results

### Patient characteristics

Among 4981 children, a total of 1631 children (32.7%), including 842 boys and 779 girls, were available for analysis in the present study. The characteristics of the mothers and their children who were available for analysis in this study were compared to those of other mothers and children who were not available for this analysis in Table 2. 2D/4D and PSAI were both derived from the following participants; older mothers, a higher annual household income, higher educational level, and fewer smokers during pregnancy. These factors did not affect 2D/4D or PSAI in the present study. Furthermore, no significant differences were observed in the gender, birth weight, or gestational age of children.

**Table 2:** Participant characteristics.

		2D/4D(+) and PSAI(+)		2D/4D(-) or PSAI(-)			
		n (%)	Mean ± SD	n (%)	Mean ± SD		p-value
Maternal characteristics							
Age at delivery (years old)		1631	30.7 ± 4.3	3344		29.6 ± 4.6	**
Pre-pregnancy BMI (m^2^/kg)		1577	20.9 ± 3.3	3242		21.1 ± 4.3	
Parity	Primiparous	733 (46.2)		1319 (40.8)			**
	Multiparous	843 (53.8)		1913 (59.2)			
Annual household income (million yen per year)	<5	870 (61.8)		1837 (67.7)			**
	≥5	537 (38.2)		878 (32.3)			
Educational level (years)	≤12	604 (38.5)		1705 (52.5)			**
	≥13	966 (61.5)		1542 (47.5)			
Smoking during pregnancy	Non-smoker	1173 (81.9)		1883 (71.3)			**
	Smoker	260 (18.1)		758 (28.7)			
Alcohol consumption during pregnancy	Non-drinker	803 (77.1)		1699 (78.0)			
	Drinker	239 (22.9)		480 (22.0)			
Infant characteristics							
Gender	Males	852 (52.2)		1683 (50.3)			
	Females	779 (47.8)		1662 (49.7)			
Birth weight (g)		1625	3043.4 ± 388.8	3337		3040.0 ± 391.6	
Gestational age (weeks)		1584	38.8 ± 1.4	3288		38.8 ± 1.4	

**: p<0.01

SD: standard deviation

### PSAI

Composite, masculine, and feminine scores in PSAI were shown in Table 3 for boys and girls. Composite scores in PSAI were significantly higher in boys than in girls (p<0.0001). While masculine scores were significantly higher in boys than in girls, feminine scores were significantly lower in boys.

**Table 3:** Summary of PSAI.

Score	n	Mean +/-SD	Min	25%	50%	75%	Max
Boys							
Composite	852	59.7 +/- 8.3^***^	19.6	53.7	60.3	65.8	82.3
Masculinity	852	34.9 +/- 7.0^***^	16	30	35	39	58
Femininity	852	23.4 +/- 4.9^***^	11	20	23	26	51
Girls							
Composite	779	29.3 +/- 9.0	4.2	22.9	28.4	35.0	76.8
Masculinity	779	24.3 +/- 5.8	12	20	24	28	50
Femininity	779	40.5 +/- 6.2	12	37	41	45	57

***: p<0.0001 significant difference between boys and girls

### 2D/4D

In all right hand, left hand, and mean value, 2D/4D was significantly lower in boys than in girls: 93.5+/-3.7 vs. 94.9+/-3.7 in the right hand, 93.0+/-3.8 vs. 94.4+/-3.6 in the left hand, and 93.2+/-3.2 vs. 94.7+/-3.2 as the mean value (p<0.0001, respectively). 2D/4D exhibited a normal distribution in all right hands, left hands, and mean values. The mean 2D/4D value in both hands was used to investigate its relationship with PSAI as a representative value of each participant.

### Effect of brothers or sisters on PSAI (Fig 1)

**Fig 1:**
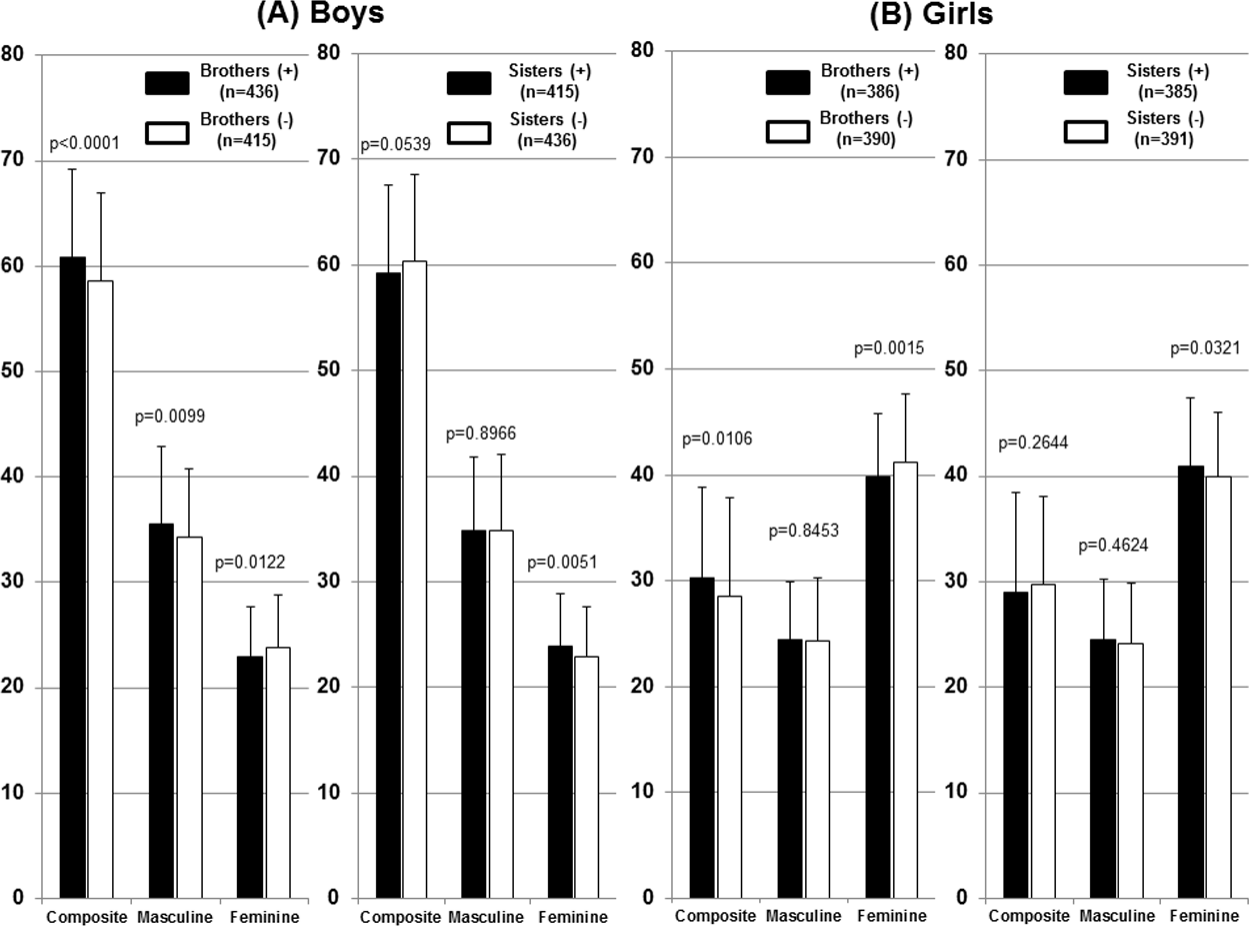
Effects of brothers or sisters on PSAI. (A) Boys. While the existence of brothers increased boy-typical behaviors and decreased girl-typical behaviors, that of sisters increased girl-typical behaviors in boys. (B) Girls. Girls with brothers exhibited fewer girl-typical behaviors due to the existence of brothers. Meanwhile, the existence of sisters increased girl-typical behaviors, even in girls.

### Boys

Composite and masculine scores were significantly higher, whereas feminine scores were significantly lower in boys with brothers than in boys with no brothers. Thus, the existence of brothers increased boy-typical behaviors and decreased girl-typical behaviors. Regarding the existence of sisters, only feminine scores were higher in boys with sisters, which indicated that the existence of sisters increased girl-typical behaviors in boys.

### Girls

The existence of brothers increased composite scores and significantly decreased feminine scores. Girls with brothers displayed fewer girl-typical behaviors due to the existence of brothers. On the other hand, the existence of sisters only increased feminine scores, which indicated that the existence of sisters led to more girl-typical behaviors, even in girls.

### Relationship between PSAI and 2D/4D (Table 4)

An analysis of Spearman’s rank-order correlation revealed that the composite scores of PSAI negatively correlated with the 2D/4D composite scores of PSAI in boys, whereas no correlation was observed in girls. Masculine scores negatively correlated with 2D/4D in boys and girls, whereas no correlation was noted in feminine scores.

**Table 4:** Analyses of the relationship between 2D/4D and PSAI in Spearman’s rank-order correlation.

PSAI		Composite	Masculine	Feminine
	n	r_s_	r_s_	r_s_
Boys	852	-0.130 **	-0.126 **	0.024
Girls	779	-0.064	-0.095**	-0.008

**: p<0.01

### Multivariate analysis of PSAI (Tables 5, 6A and 6B)

A multiple linear regression analysis revealed an inverse relationship between the composite scores of PSAI and 2D/4D in boys, but not in girls. The existence of brothers also affected composite scores in boys only. An inverse correlation was observed between masculine scores and 2D/4D in boys and girls. The existence of brothers also affected masculine scores in boys only. However, feminine scores were not associated with 2D/4D in boys or girls. The existence of sisters for boys or brothers for girls significantly affected feminine scores.

**Table 5:** Analyses of the relationship between 2D/4D and PSAI in a multiple linear regression analysis.

PSAI		Composite		Masculine		Feminine	
	n	B (95%CI)	R^2^	B (95%CI)	R^2^	B (95%CI)	R^2^
Boys	786	-0.129** (-0.520, -0.158)	0.041	-0.129** (-0.444, -0.133)	0.028	-0.013 (-0.088, 0.126)	0.021
Girls	704	-0.038 (-0.321, 0.103)	0.017	-0.088* (-0.293, -0.025)	0.017	-0.030 (-0.207, 0.087)	0.019

Covariates:birth weight, maternal smoking during pregnancy, maternal alcohol consumption during pregnancy, brothers, sisters

*: p<0.05

**: p<0.01

**Table 6:** Analysis of the relationship between PSAI and the existence of brothers or sisters in a multiple linear regression analysis.

**A. Existence of brothers**							
PSAI		Composite		Masculine		Feminine	
	n	B (95%CI)	R^2^	B (95%CI)	R^2^	B (95%CI)	R^2^
Boys	786	0.134** (0.496, 1.694)	0.041	0.102** (0.196, 1.225)	0.028	-0.059 (-0.639, 0.069)	0.021
Girls	704	-0.072 (-0.078, 1.389)	0.017	0.010 (-0.407, 0.518)	0.017	-0.086* (-0.148, -0.034)	0.019
**B. Existence of sisters**							
PSAI		Composite		Masculine		Feminine	
	n	B(95%CI)	R^2^	B(95%CI)	R^2^	B(95%CI)	R^2^
Boys	786	-0.031 (-0.855, 0.347)	0.041	0.041 (-0.229, 0.803)	0.028	0.108** (0.163, 0.873)	0.021
Girls	704	-0.021 (-0.920, 0.548)	0.017	0.025 (-0.321, 0.605)	0.017	0.050 (-0.197, 0.819)	0.019

Covariates: birth weight, maternal smoking during pregnancy, maternal alcohol consumption during pregnancy, brothers, sisters

*: p<0.05

**: p<0.01

## Discussion

The relationship between gender-role play behavior in PSAI and 2D/4D in school-aged children was investigated in the present study. We herein demonstrated that 2D/4D was significantly lower in boys than in girls, which was consistent with previous findings[12, 13]. Composite scores in PSAI were significantly higher in boys than in girls, as previously reported^18 19^. Masculine scores were significantly higher in boys than in girls, while feminine scores were significantly lower in boys. Although the existence brothers or sisters also affected boy-typical or girl-typical behaviors in PSAI, a multiple linear regression analysis revealed an inverse association between the composite scores of PSAI and 2D/4D in boys, but not in girls, and an inverse correlation was observed in the masculine scores of PSAI with 2D/4D in boys and girls. However, feminine scores were not associated with 2D/4D in boys or girls. Thus, the present study revealed that prenatal androgen exposure derived from 2D/4D may have influenced masculine-typical dimorphic brain development and behavior in school-aged children.

The extent of prenatal androgen exposure affected differentiation to male-typical external and internal genitalia. The sexual difference observed in 2D/4D has already been established during early prenatal development under the influence of sex hormones[23, 24], and has also been attributed to the prenatal hormonal environment, such as exposure to higher levels of androgens and some other gonad-specific hormones[25] through androgen receptors, which are located in fetal cartilaginous tissue[14]. Therefore, 2D/4D has been used as an easily measurable and stable anthropometric index of prenatal androgen exposure. Another cohort study in our study group showed that 2D/4D was already smaller in boys than in girls at birth (data not shown). Furthermore, we previously reported that 2D/4D in school-aged children was significantly lower in boys than in girls and was also affected by prenatal Leydig cell function in males[13].

Anatomical studies showed male-typical characteristics in the human brain, such as a larger and heavier brain, larger cerebrum, smaller frontal and medial paralimbic cortices, and larger frontomedial cortex. These anatomical sex dimorphisms are also considered to be affected by prenatal androgen exposure[26]. Regarding cognitive functions, previous studies indicated that the hormone environment during early gestation, particularly androgen exposure, also affected behavioral dimorphism between boys and girls. In humans, clinical studies previously reported behavioral changes in people with atypical sex hormone exposure. Previous studies showed that girls with CAH, who were exposed to excessive amounts of androgens during gestation, showed more boy-typical behaviors and personality[3–6]. Children were also shown to exhibit more male-typical behaviors if their mothers took androgenic progestin during pregnancy. On the other hand, children with CAIS, who do not respond to androgens despite normally functioning testes, show female-typical behaviors[7, 8]. Furthermore, fewer male-typical behaviors have been reported in children whose mothers were prescribed anti-androgenic hormones during pregnancy[27–29].

In the present study, we detected an inverse association between the composite scores of PSAI and 2D/4D in boys, but not in girls. Furthermore, an inverse correlation was observed in the masculine scores of PSAI with 2D/4D in boys and girls, whereas feminine scores were not associated with 2D/4D in boys or girls. These results indicated that prenatal androgen exposure influenced masculine-typical dimorphic brain development and behavior in school-aged children.

In addition to the hormonal environment during the prenatal period, the postnatal environment may also affect physical development and behavioral sexual dimorphism in children. Regarding the hormonal environment after birth, testosterone levels in urine were shown to be higher in boys than in girls between 1 month and 6 months of age, but declined to baseline levels by approximately 6 months of age[30]. Testosterone levels were also found to be lower before and after this period until puberty. Other hormones, such as estradiol, LH, and FSH, have also been shown to transiently increase in the first year of life. This transient surge in hormones is known as minipuberty. Knickmeyer et al reported that testosterone levels in the neonatal period, particularly in the first 2 years of life, may modulate 2D/4D in boys[31]. This minipuberty in the early period after birth has also been shown to influence somatic development[32]. In the sexual dimorphism of behaviors, the results of PSAI revealed that testosterone levels during early infancy may also affect neurobehavioral sexual dimorphism[30]; however, this was not measured in the present study.

In addition to the hormonal environment, social factors are known to affect dimorphic brain development and behavior in children. In a previous study using PSAI in adopted children with different types of parents, such as lesbian, gay, and heterosexual parents, gender-typed play behavior was affected by parents, suggesting that boys of lesbian mothers were less masculine in their play behavior than boys of gay fathers and heterosexual parents[33]. PSAI in the present study revealed that the existence of brothers and sisters also affected gender-typed play behavior. In boys, the existence of brothers increased boy-typical behaviors, whereas the existence of sisters increased girl-typical behaviors. A multivariate analysis revealed that girls with brothers exhibited fewer girl-typical behaviors due to the existence of brothers. Further studies are needed in order to determine whether other social factors, e.g. friends and teachers, also affect dimorphic brain development and behavior in childhood.

The present study identified possibility that the prenatal hormonal environment could be one of the important factors affecting sexually dimorphic brain development and behavior as well as physical changes. However, considering other factors that influence cognitive functions between boys and girls, there were still some limitations in the present study. We did not measure hormone levels during the prenatal period in the present study. Therefore, we did not concretely demonstrate that the prenatal hormonal environment affected dimorphic brain development and behavior in school-aged children. Additionally, although we revealed that 2D/4D was associated with masculine-typical dimorphic brain development and behavior, we did not exclude the postnatal effects of hormones in 2D/4D, particularly in minipuberty. Therefore, longitudinal studies of 2D/4D, which may reveal the real meaning of 2D/4D in the hormonal environment, are warranted in the future. Furthermore, PSAI was originally developed for preschool children, but was used for school-aged children in the present study. PSAI also cannot accurately reflect behavioral aspects in children nowadays because they often play electronic games and with their smartphones. Thus, our study may only have identified one aspect of sexually dimorphic brain development and behavior. A new questionnaire needs to be developed based on the current generation of children.

## Conclusions

The present study revealed possibility that the prenatal hormonal environment, particularly androgen exposure during early gestation, could be one of the important factors influencing masculine-typical dimorphic brain development and behavior in school-aged children. Further, social factors, such as the existence of brother or sisters, also could affect gender role behaviors in childhood.

## Abbreviations

2D/4D: second-to-fourth digit ratio
PSAI: Pre-school Activities Inventory
CAH: congenital adrenal hyperplasia
CAIS: complete androgen insensitivity syndrome
